# Letter to Editor “Melatonin as a Novel Drug to Improve Cardiac Function and Quality of Life in Heart Failure Patients: A Systematic Review and Meta‐Analysis”

**DOI:** 10.1002/clc.70325

**Published:** 2026-04-27

**Authors:** Neda Roshanravan, Sina Pakkhesal, Faezeh Tarighat, Samad Ghaffari

**Affiliations:** ^1^ Cardiovascular Research Center Tabriz University of Medical Sciences Tabriz Iran; ^2^ Faculty of Pharmacy Tabriz University of Medical Sciences Tabriz Iran

## Conflicts of Interest

The authors declare no conflicts of interest.

Dear Editor,

We want to thank the authors of *“Melatonin as a Novel Drug to Improve Cardiac Function and Quality of Life in Heart Failure Patients: A Systematic Review and Meta‐Analysis”* [1] for their rigorous evaluation of melatonin as an adjunct therapy in heart failure (HF). Insomnia impacts around 9.7% of patients with HF and is a significant risk factor for negative cardiac outcomes. Effectively managing insomnia is crucial, but it often becomes challenging due to the complexity of polypharmacy and existing comorbidities. Addressing this issue is essential for improving patient care and outcomes. Recently, melatonin has emerged as a safe and moderately effective adjuvant agent, making it a highly appealing option for those seeking complementary treatments. However, the findings reveal a complex scenario. While melatonin improved quality of life (*p* < 0.05), it did not significantly affect ejection fraction or NYHA class (*p* ≥ 0.05) across four small randomized trials (*n* = 35–85, ≤ 24 weeks). This raises the question of whether benefits are primarily symptomatic rather than disease‐modifying.

Recent concerns regarding long‐term safety further complicate interpretation. Observational data by Nnadi et al. link prolonged melatonin use (≥ 1 year) to increased risks of HF incidence (HR 1.89), hospitalization (HR 3.44), and all‐cause mortality (HR 2.09) [2]. We propose that chronic desensitization of melatonin receptors may provide a mechanistic explanation for this discrepancy. Sustained exposure could disrupt neuro‐hormonal, cellular, and metabolic pathways, potentially leading to cardiovascular decline. In this realm, several mechanisms have been suggested, including.

First, long‐term melatonin use (LMU) can disrupt the balance of neuro‐hormones in the body. Normally, melatonin helps lower blood pressure (BP) by acting on MT2 receptors in the suprachiasmatic nucleus (SCN), which reduces sympathetic nervous activity. However, with long‐term use, these receptors can become desensitized and internalized, making the SCN less responsive to melatonin. This desensitization can disrupt the normal nighttime drop in BP, resulting in a “non‐dipper” hypertensive state. In this condition, the heart experiences continuous pressure, which increases the risk of cardiac remodeling and HF. Additionally, the desensitization of melatonin receptors in the nodose ganglia may reduce baroreflex sensitivity and hinder the body's ability to regulate BP autonomously [3].

Second, LMU may weaken the heart's cellular defense mechanisms. Recent research shows that melatonin, through MT1 receptors, suppresses the chaperone protein FKBP4. This traps stress‐activated glucocorticoid receptors (GR) in the cytoplasm, preventing their entry into the nucleus, where they can induce mitochondrial dysfunction and apoptosis. In the pro‐inflammatory state of insomnia, cardiomyocyte MT1 receptor desensitization enables unchecked glucocorticoid‐mediated damage, accelerating the loss of contractile cells and cardiac energy starvation, which is critical to HF progression [4].

Finally, melatonin can inhibit insulin secretion, which is appropriate during nocturnal fasting. However, LMU, especially when taken in the evening near meals, can impair glucose tolerance. Over the years, this metabolic disruption can promote suboptimal glycemic regulation or progression of type 2 diabetes—a leading cause of HF [5].

**Figure 1:**
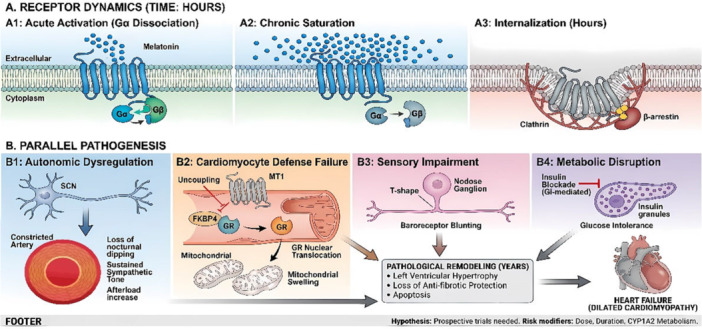
Hypothesized convergence of neuro‐hormonal, cellular, and metabolic mechanisms linking chronic melatonin supplementation to heart failure. Chronic exposure to supraphysiological melatonin levels desensitizes and internalizes MT2 receptors in the suprachiasmatic nucleus (SCN) and nodose ganglia. This leads to the loss of the healthy nocturnal blood pressure dip and reduces baroreflex sensitivity, increasing the hemodynamic workload on the heart. At the cardiomyocyte level, prolonged stimulation desensitizes MT1 receptors, removing the epigenetic suppression of FKBP4. As a result, stress‐activated glucocorticoid receptors (GR) translocate to the nucleus, causing mitochondrial dysfunction and apoptosis. Simultaneously, evening melatonin intake inhibits insulin secretion, impairing glucose tolerance and promoting type 2 diabetes. These pathways work together to promote pathological cardiac remodeling and heart failure. Hypothesized mechanisms linking chronic supraphysiological melatonin exposure to heart failure. (A) The temporal dynamics of the melatonin MT2 receptor are shown, from (A1) acute G‐protein activation to (A3) β‐arrestin‐mediated internalization. Chronic melatonin desensitizes MT1/MT2 receptors, initiating parallel pathogenic cascades (B). In the nervous system, this leads to autonomic dysregulation (B1) and blunted baroreflexes (B3), increasing cardiac afterload. At the cardiomyocyte level (B2), MT1 desensitization enables GR‐mediated mitochondrial dysfunction and cell apoptosis. Concurrently, melatonin‐induced inhibition of insulin secretion causes metabolic disruption (B4). Together, these pathways drive pathological remodeling and heart failure.

We propose a vicious cycle: LMU desensitizes pathways, leading to sustained hemodynamic stress (Pathway 1), impacting a weakened heart by the loss of intrinsic cellular defenses against stress hormones (Pathway 2), exacerbated by metabolic dysfunction (Pathway 3) (Figure 1). This model aligns with epidemiological data that a strong endogenous melatonin rhythm is linked to healthier nighttime BP, framing the problem not as melatonin being harmful, but as disrupting a finely tuned endogenous system [6]. Lastly, patients with inadequately treated insomnia may be more likely to use melatonin—typically recommended for short‐term use—for extended periods. This persistent insomnia itself, rather than LMU, could contribute to the development of HF [7].

While the cardioprotective effects of melatonin are well‐known, recent studies raise concerns about potential harm from chronic and high‐dose use in individuals with insomnia. The proposed mechanisms advocate for long‐term clinical trials to assess the cardiovascular efficacy and safety of supplements, particularly melatonin‐receptor agonists such as ramelteon, which are gaining popularity. In the interim, clinical practice should focus on administering the lowest effective dose for the shortest duration possible.

## Funding

The authors have nothing to report.

## Ethics Statement

The authors have nothing to report.

## Consent

The authors have nothing to report.

## Data Availability

The authors have nothing to report.
